# 2802. Frequency, Antimicrobial Susceptibility, and Molecular Characterization of Carbapenem-Resistant Enterobacterales Stratified by United States Census Divisions: Results from the INFORM Program (2018-2022)

**DOI:** 10.1093/ofid/ofad500.2413

**Published:** 2023-11-27

**Authors:** Helio S Sader, John H Kimbrough, Timothy Doyle, Cecilia G Carvalhaes, Mariana Castanheira

**Affiliations:** JMI Laboratories, North Liberty, Iowa; JMI Laboratories, North Liberty, Iowa; JMI Laboratories, North Liberty, Iowa; JMI Laboratories, North Liberty, Iowa; JMI Laboratories, North Liberty, Iowa

## Abstract

**Background:**

Recently approved β-lactamase inhibitor combinations (BLIs), such as ceftazidime-avibactam (CAZ-AVI) and meropenem-vaborbactam (MEM-VAB), have demonstrated a broad spectrum of activity against carbapenem-resistant Enterobacterales (CRE) from US hospitals, but resistance may emerge with the increasing use of these compounds. Aztreonam-avibactam (ATM-AVI) has shown potent activity against CRE, including MBL producers, and is under clinical development. We evaluated the activity of ATM-AVI and comparators against CREs from US hospitals.

**Figure:**
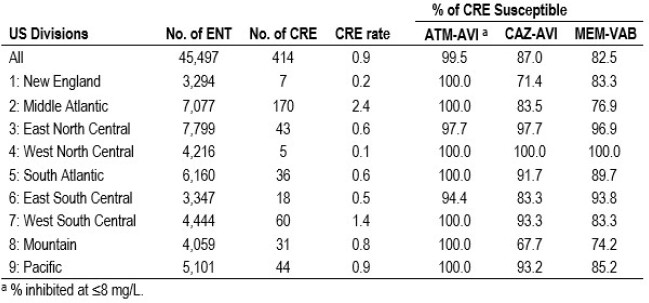

**Methods:**

45,497 Enterobacterales (ENT) isolates were consecutively collected from 79 US medical centers (36 states) and susceptibility tested by CLSI broth microdilution. ATM-AVI was tested with AVI at a fixed 4 mg/L and a susceptible (S) breakpoint of ≤ 8 mg/L was applied for comparison. CRE isolates were screened for carbapenemase (CPE) by whole genome sequencing.

**Results:**

ATM-AVI inhibited > 99.9% of ENT at ≤4 mg/L and only 4 isolates (< 0.01%) showed ATM-AVI MICs > 8 mg/L. CAZ-AVI (MIC_50/90_, 0.12/0.25 mg/L) and MEM-VAB (MIC_50/90_, 0.03/0.06 mg/L) were active against 99.9% and 99.8% of ENT isolates, respectively. CRE rates varied from 0.2% (New England [NE]) to 2.4% (Middle Atlantic; Table). ATM-AVI was active (MIC ≤ 8 mg/L) against 99.5% (412/414) of CREs, whereas susceptibility to CAZ-AVI and MEM-VAB were lowest in the Mountain [MO] division (67.7% and 74.2%, respectively) and highest (100.0%) in West North Central. ATM-AVI retained activity against ENT non-S to CAZ-AVI and/or MEM-VAB (n=73; MIC_50/90_, 0.25/2 mg/L; 98.6% inhibited at ≤8 mg/L). KPC was the most common CPE (65.5% of CREs), followed by NDM (8.2%) and OXA-48–like (3.6%). A CPE gene was not observed in 20.8% of CREs. The occurrence of KPC among CREs varied from 14.3% (1/7; NE) to 77.8% (14/18; East South Central [ESC]); whereas the frequency of metallo-β-lactamases (MBLs) ranged from ≤ 3.0% (South Atlantic, East North Central, ESC and Pacific) to 19.4% (6/31) in MO and 42.9% (3/7) in NE.

**Conclusion:**

ATM-AVI showed potent activity against CRE, including MBL producers, from all US Census Divisions. Resistance to CAZ-AVI and MEM-VAB among CRE was observed in the NE and MO Census Divisions due to increasing occurrence of MBL-producing isolates.

**Disclosures:**

**Helio S. Sader, MD, PhD, FIDSA**, AbbVie: Grant/Research Support|Basilea: Grant/Research Support|Cipla: Grant/Research Support|Paratek: Grant/Research Support|Pfizer: Grant/Research Support|Shionogi: Grant/Research Support **John H. Kimbrough, PhD**, AbbVie: Grant/Research Support|Basilea: Grant/Research Support|Pfizer: Grant/Research Support|Shionogi: Grant/Research Support **Timothy Doyle, MS**, AbbVie: Grant/Research Support **Cecilia G. Carvalhaes, MD, PhD**, AbbVie: Grant/Research Support|bioMerieux: Grant/Research Support|Cipla: Grant/Research Support|CorMedix: Grant/Research Support|Melinta: Grant/Research Support|Pfizer: Grant/Research Support **Mariana Castanheira, PhD**, AbbVie: Grant/Research Support|Basilea: Grant/Research Support|bioMerieux: Grant/Research Support|Cipla: Grant/Research Support|CorMedix: Grant/Research Support|Entasis: Grant/Research Support|Melinta: Grant/Research Support|Paratek: Grant/Research Support|Pfizer: Grant/Research Support|Shionogi: Grant/Research Support

